# A structural role for the PHP domain in *E. coli* DNA polymerase III

**DOI:** 10.1186/1472-6807-13-8

**Published:** 2013-05-14

**Authors:** Tiago Barros, Joel Guenther, Brian Kelch, Jordan Anaya, Arjun Prabhakar, Mike O’Donnell, John Kuriyan, Meindert H Lamers

**Affiliations:** 1 Howard Hughes Medical Institute, Department of Molecular and Cell Biology and Department of Chemistry, University of California, Berkeley, CA 94720, USA; 2 Laboratory of DNA Replication, Howard Hughes Medical Institute, The Rockefeller University, New York, NY 10021, USA; 3 Physical Biosciences Division, Lawrence Berkeley National Laboratory, Berkeley, CA 94720, USA; 4 Present address: MRC Laboratory of Molecular Biology, Hills Road, Cambridge CB2 0QH, UK

**Keywords:** DNA polymerase III, DNA replication, PHP domain, Proofreading exonuclease

## Abstract

**Background:**

In addition to the core catalytic machinery, bacterial replicative DNA polymerases contain a Polymerase and Histidinol Phosphatase (PHP) domain whose function is not entirely understood. The PHP domains of some bacterial replicases are active metal-dependent nucleases that may play a role in proofreading. In *E. coli* DNA polymerase III, however, the PHP domain has lost several metal-coordinating residues and is likely to be catalytically inactive.

**Results:**

Genomic searches show that the loss of metal-coordinating residues in polymerase PHP domains is likely to have coevolved with the presence of a separate proofreading exonuclease that works with the polymerase. Although the *E. coli* Pol III PHP domain has lost metal-coordinating residues, the structure of the domain has been conserved to a remarkable degree when compared to that of metal-binding PHP domains. This is demonstrated by our ability to restore metal binding with only three point mutations, as confirmed by the metal-bound crystal structure of this mutant determined at 2.9 Å resolution. We also show that Pol III, a large multi-domain protein, unfolds cooperatively and that mutations in the degenerate metal-binding site of the PHP domain decrease the overall stability of Pol III and reduce its activity.

**Conclusions:**

While the presence of a PHP domain in replicative bacterial polymerases is strictly conserved, its ability to coordinate metals and to perform proofreading exonuclease activity is not, suggesting additional non-enzymatic roles for the domain. Our results show that the PHP domain is a major structural element in Pol III and its integrity modulates both the stability and activity of the polymerase.

## Background

The DNA polymerases at the core of every bacterial replisome belong to the C-family of DNA polymerases [1]. All members of this family contain a set of four domains that are organised within a single polypeptide in the following order: Polymerase and Histidinol Phosphatase (PHP), Palm, Thumb and Fingers. While these four domains always appear in the same order, the DNA polymerase III (Pol III) and DNA polymerase C (Pol C) subfamilies can be distinguished within the C-family of DNA polymerases, depending on the arrangement of additional accessory domains, such as the OB-fold domain that binds single-stranded DNA [2-4]. Neither Pol III nor Pol C share detectable sequence homology with other DNA polymerases, including bacterial DNA polymerases such as Pol I and Pol II and the eukaryotic replicative DNA polymerases ϵ and δ.

The crystal structures of *Escherichia coli* (*E. coli*) Pol III [5], *Thermus aquaticus* (*T. aquaticus*) Pol III [6] and *Geobacillus kaustophilus* (*G. kaustophilus*) Pol C [7] have shown that the active sites of these polymerases are structurally related to that of human DNA polymerase β, an atypical DNA polymerase that is involved in base excision repair and belongs to the X-family of DNA polymerases. This is surprising, as X-family polymerases are typically slow and exhibit low fidelity and processivity, in contrast to the high-fidelity replicative C-family polymerases, which are amongst the fastest polymerases known.

Pol III and Pol C polymerases are also unique in that they contain a Polymerase and Histidinol Phosphatase (PHP) domain that is not found in other polymerases, except for some bacterial Pol X family members [8]. The PHP domain in Pol III and Pol C is a barrel-shaped domain located at the side of the polymerase, near the Thumb domain [5,6]. The active site of typical PHP domains is a shallow cavity located at the top of the barrel-shaped domain, usually consisting of seven β-strands that provide most of the residues that coordinate the catalytic metals. Several crystal structures of PHP domains have been determined. These include PHP domains that are not part of a larger protein: *E. coli* YcdX [9], *Thermus thermophilus* (*T. thermophilus*) histidinol phosphate phosphatase (ppPHP) [10] and tm0559 from *Thermatoga maritima* (*T. maritima*) (pdb code 2ANU); or those that are part of polymerases: *E. coli* Pol III [5], *T. aquaticus* Pol III [6], *G. kaustophilus* Pol C [7] and *Deinococcus radiodurans* (*D. radiodurans*) Pol X [11].

The role of the PHP domain in Pol III and Pol C polymerases remains unclear. When first identified in sequence alignments, the PHP domain in Pol III/Pol C was hypothesized to act as a pyrophosphatase, removing the by-product of DNA synthesis in order to drive the polymerization reaction in the direction of DNA synthesis [8]. However, no such activity has been detected as yet for a polymerase PHP domain. Instead, the PHP domains of *T. thermophilus* Pol III and *T. aquaticus* Pol III, which have a complete set of metal-coordinating residues and have been shown to bind metals, have exonuclease activity [12,13], which presumably serves to proofread newly synthesized DNA. Likewise, the PHP domains of Pol X from both *Bacillus subtilis* (*B. subtilis*) [14] and *T. thermophilus*[15] have exonuclease activity. In contrast, no exonuclease activity could be detected for the PHP domain of *G. kaustophilus* Pol C [7].

The invariable presence of the PHP domain in all C-family polymerases, even those lacking exonuclease activity, suggests that this domain must play an essential, yet non-enzymatic, role in maintaining the activities of these polymerases. We show, using sequence analysis, that the loss of metal-coordinating residues in the Pol III PHP domain is correlated with the presence in bacterial genomes of a protein homologous to the *E. coli* Pol III proofreading exonuclease ϵ subunit. Despite the apparent loss of catalytic function, the structural scaffold of the PHP domain has been conserved to a remarkable degree. This observation is strongly supported by our ability to restore metal binding to the *E. coli* Pol III PHP domain by introducing only three point mutations. We further show that the structural integrity of the PHP domain is important for the stability and activity of the *E. coli* Pol III.

## Results and discussion

### A complete set of metal-coordinating residues is not universally conserved in DNA polymerase PHP domains

**Figure 1:**
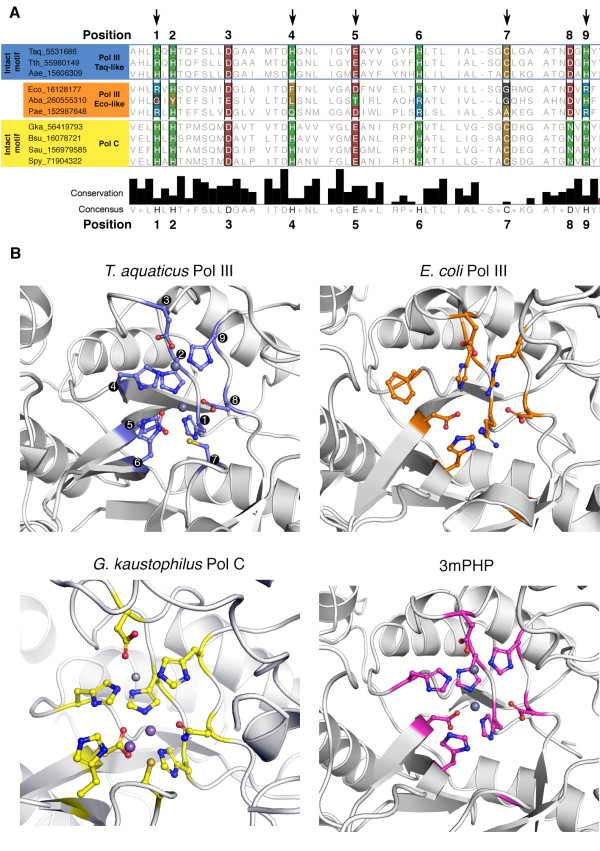
**PHP domain metal-coordinating residues are not conserved.** (**A**) Sequence alignment of C-family DNA polymerase PHP domains. The figure shows a selected set of sequences from our larger (47-sequence) alignment. Only sequences of polymerases that have been structurally or biochemically characterized were selected. For each polymerase the GI number and subtype within the C-family is indicated. For the conservation score diagram, the height of the bars is proportional to the conservation of the residues in our large alignment of C-family DNA polymerase sequences, as determined according to [17]. Black arrows at the top indicate the positions of variation in *E. coli*. (**B**) PHP domain cleft of C-family DNA polymerases*.* Metal-binding residues (or their substitutes in mutated PHP domains) are shown in ball and stick representation. Phosphate ions in *E. coli* Pol III and *G. kaustophilus* Pol C have been omitted for clarity.

Aravind and Koonin identified the conservation of a PHP domain in all C-family DNA polymerases [8]. In the same study, the authors recognized that in some bacteria, including *E. coli*, not all PHP metal-coordinating residues are conserved, and they predicted that these variant PHP domains would be enzymatically inactive. Extending this study, we selected a set of 47 C-family DNA polymerase sequences and aligned them using MAFFT [16] (Alignment in Additional file 1). We find that the two types of PHP domains (those carrying an intact set of metal-coordinating residues and those variant ones that have an incomplete set of these residues) appear in both Pol III and Pol C and are widespread across multiple phyla. In PHP domains that bind metals, nine conserved residues coordinate three metal ions (Figure 1). In the PHP domain of *E. coli* Pol III, five of these metal-coordinating residues are replaced by residues incompatible with this function, and the domain was therefore thought to not bind metal ions [8]. This was indeed confirmed by its crystal structure [5]. Given that the vast majority of PHP domains appears to bind metals, it is likely that the ancestral Pol III and Pol C PHP domains had metal-binding capability, and that this function has been lost in some bacteria during evolution. In this report, we refer to PHP domains that have an incomplete set of metal-coordinating residues as variant PHP domains.

Examining the sequence of variant PHP domains in detail, we find that the histidines at positions 4, 6 and 9 are the most frequently lost (in *E.* coli the residues at the corresponding positions are Phe 44, His 83 and Arg 203; Figure 1, Table 1). Including those three histidines, the most frequent replacement at six of the nine metal coordinating positions is substitution of the canonical residue for an arginine. This can be seen as a structurally conservative replacement, as the positivly charged metal ion is replaced by a positively charged sidechain. This can occur without significant structural distortion, as illustrated by the replacement of a metal ion in a mutant D-xylose isomerase by a lysine residue that is substituted for a glutamate that normally coordinates a metal [18]. The cysteine at position 7 is most often replaced by glycine or alanine (G134 in *E. coli*), and in roughly 20% of the analysed sequences the loop containing this residue is absent. Interestingly, our alignment also shows that the residues immediately adjacent to those responsible for metal-binding are generally more conserved than the metal-binding residues themselves.

**Table 1:** Conservation of the nine residues required for metal binding in PHP domains

		**Metal binding residue**									**Metal**		
	**PDB**	**1**	**2**	**3**	**4**	**5**	**6**	**7**	**8**	**9**	**Me1**	**Me2**	**Me3**
**Consensus**		**H**	**H**	**D/H**	**H**	**E**	**H**	**C/H**	**D/N**	**H**	**Zn**		
Eco Pol III	2hnh	R_10_	H_12_	D_19_	F_44_	D_69_	H_83_	G_134_	D_201_	R_203_	R_203_	R_10_	-
3mPHP	4jom	**H**_**10**_	"	"	**H**_**44**_	"	"	"	"	**H**_**203**_	Zn	Zn	-
4mPHP	n.a.	**H**_**10**_	"	"	**H**_**44**_	"	"	**C**_**134**_	"	**H**_**203**_	n.a.	n.a.	n.a.
5mPHP	n.a.	**H**_**10**_	"	"	**H**_**44**_	**E**_**69**_	"	**C**_**134**_	"	**H**_**203**_	n.a.	n.a.	n.a.
Taq Pol III	2hpi	H_11_	H_13_	D_20_	H_47_	E_72_	H_95_	C_145_	D_212_	H_214_	Zn	Zn	-
Gka PolC	3f2d	H_346_	H_348_	D_355_	H_380_	E_405_	H_620_	C_670_	N_743_	H_745_	Zn	Mn	Mn

Mutations introduced in the PHP domain of Eco Pol III are indicated in bold. M1, M2, M3 are metal positions going from outside to inside of the PHP ligand binding site.

The PHP active site in *E. coli* Pol III has 5 replacements compared to the consensus sequence (Figure 1). In addition to the three histidines and the cysteine at position 7 mentioned previously, a glutamate to aspartate replacement at position 5 (Asp 69) is present. Indeed, in our hands the *E. coli* Pol III subunit does not show any nuclease activity (see below). The lack of activity by the *E. coli* PHP domain has been predicted [8]. It is surprising, however, that the *G. kaustophilus* PHP domain is also inactive [7] as it presents an almost intact active site that has been shown to bind metals. The only replacement in this active site is an aspartate to asparagine substitution at position 8. It is likely that additional residues not directly involved in metal coordination are also necessary for robust exonuclease activity.

### The presence of variant PHP domains in bacterial replicative polymerases is correlated with the presence of separate proofreading exonucleases

**Figure 2:**
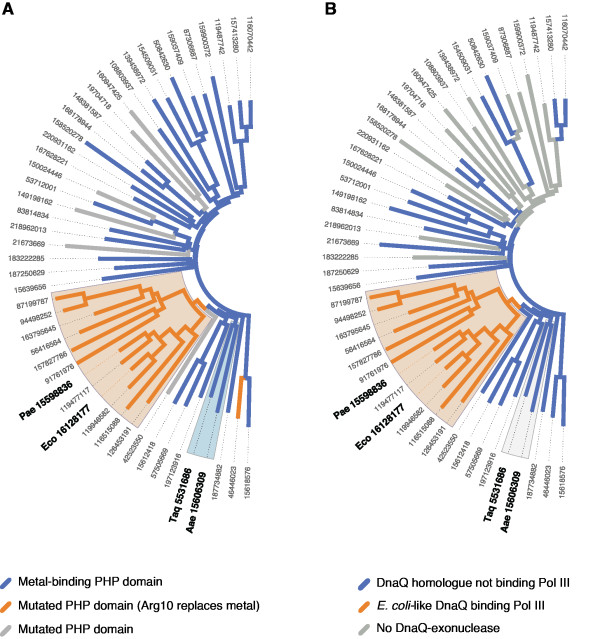
**A separate proofreading subunit coevolved with variant PHP domains.** The trees were constructed using 50 selected sequences from our 47-sequence alignment of C-family DNA polymerases and 72 exonuclease sequences. Numbers indicate the GenInfo Identifier of the polymerase sequences. Two clades, corresponding to (1) α-, β- or γ-proteobacteria and (2) *Thermus aquaticus* and *Aquifex aeolicus* are shaded light orange and light grey in both trees, respectively. The tree in (**B**) shows whether the species to which the polymerase sequence corresponds contains an *E. coli*-like DNA polymerase III ϵ subunit homologue or not.

Our alignment reveals that Pol III proteins with variant PHP domains mostly belong to the phylum proteobacteria, one of the major groups of bacteria. Within this phylum, our alignment shows that the PHP domains that presumably lost metal-binding capability are found in the genomes of the α-, β- and γ-proteobacteria classes, but not in δ- or ϵ-proteobacteria (Figure 2A). α-, β- and γ-proteobacteria, within which *E. coli* is included, form a monophyletic clade and are therefore likely to have evolved from a common ancestor[19]. This suggests that the loss of metal-binding and, presumably, enzymatic activity, occurred in the last common ancestor of those proteobacteria. Our analysis therefore suggests that DNA polymerases without active PHP domains must have evolved from an ancestral version in which the metal-binding capacity of the PHP domain is intact.

In *E. coli* Pol III, the proofreading exonuclease function is provided by the ϵ subunit of the DNA polymerase III holoenzyme [20]. We wondered if the presence of an ϵ subunit was a general feature of holoenzymes containing a Pol III protein with a variant PHP domain. The ϵ subunit of the DNA polymerase III holoenzyme, also known as DnaQ, is a member of the DEDD superfamily of DNAses and RNAses, which have in common a set of four strictly conserved acidic residues (DEDD) that are responsible for binding two catalytic metal ions [21]. Within the DEDD superfamily, a distinction is made between the DEDDh and DEDDy subfamilies, based on whether a fifth conserved residue is a tyrosine (DEDDy) or a histidine (DEDDh), as in the *E. coli* DNA polymerase III ϵ subunit.

From the genomes of the 47 bacterial species represented in our C-family DNA polymerase sequence alignment, which include 12 from α-, β- or γ-proteobacteria, we extracted the sequences of 72 proteins containing a DEDD exonuclease domain (sequence alignment in Additional file 2). Visualizing the relatedness of the sequences as a phylogenetic tree (Figure 2B), we observed that the *E. coli* DNA polymerase III ϵ subunit is part of a clade of 12 sequences. The sequences within this clade belong exclusively to genomes of α-, β- or γ-proteobacteria. Each species is represented by one DEDDh sequence. No other class of bacteria was represented in the clade. We also observed that all the exonucleases in the clade contain a C-terminal tail homologous to the one that the *E. coli* ϵ subunit uses to bind to its Pol III α subunit partner, and these contain multiple highly conserved residue, two of which have been demonstrated to be important for binding to the PHP domain of the polymerase (in *E. coli:* His 225, Trp 241) [22,23]. From these observations, we hypothesize that a *bona fide* ϵ subunit, which we define as being both a member of the DEDDh family of exonucleases and containing a C-terminal tail that binds to the α subunit, is only found in α-, β- and γ- proteobacteria. We stress that, while members of the DEDDh family of exonucleases are very common, few have been functionally characterized. Indeed, *E. coli* contains five such DNA exonuclease paralogs, but only its DNA polymerase III ϵ subunit is essential for viability [24]. Although our sequence analysis cannot exclude the possibility that a different DEDDh subtype may be essential for some bacteria and involved in DNA replication proofreading, we find no support for the existence of a canonical DNA polymerase III ϵ subunit outside of α-, β- or γ- proteobacteria, whose DNA polymerase III PHP domain seems to have lost metal-binding capability. Therefore, we hypothesize that the proofreading activity for Pol III is supplied by either a metal-binding PHP domain or, in α-, β- or γ-proteobacteria, by a separate protein equivalent to the DNA polymerase III holoenzyme ϵ subunit in *E. coli*.

### Re-introduction of the catalytic residues in the PHP domain of E. coli Pol III restores metal binding

Superposition of the PHP domains of *E. coli* Pol III (Eco), *T. aquaticus* Pol III (Taq) and *G. kaustophilus* Pol C (Gka) reveals a striking structural conservation of the domain. There is relatively low sequence identity between the domains of the three species: 38% over 270 residues between Eco and Taq, 26% over 270 residues between Eco and Gka and 24% over 280 residues between Taq and Gka; however the root mean square deviation of the Cα positions is only 1.20 Å (over 220 aligned atoms), 1.34 Å (over 182 aligned atoms) and 1.10 Å (over 174 aligned atoms) for the Eco/Taq, Eco/Gka and Taq/Gka superpositions, respectively.

Given that the structural similarity among the PHP domain is greater than that expected based on the level of sequence identity [25], we endeavoured to restore metal binding by reverting the variant residues to those found in canonical PHP domains. For this, we made three variants of *E. coli* Pol III with three, four or five mutations in the PHP domain, termed 3mPHP, 4mPHP, and 5mPHP, respectively (see Table 1). The first of these mutants, 3mPHP, has three histidine residues restored at positions 1, 4 and 9 (i.e. R10H, F44H, R203H). 4mPHP has an additional glycine to cysteine mutation introduced at position 7 (i.e. R10H, F44H, G134C, R203H), while 5mPHP has a fifth and final introduced mutation of Asp69 to glutamate at position 5 (i.e. R10H, F44H, D69E, G134C, R203H) to complete the canonical PHP metal-binding motif. All mutations were made in the truncated version of the polymerase (truncated after residue 917) that was used to determine the crystal structure [5].

**Figure 3:**
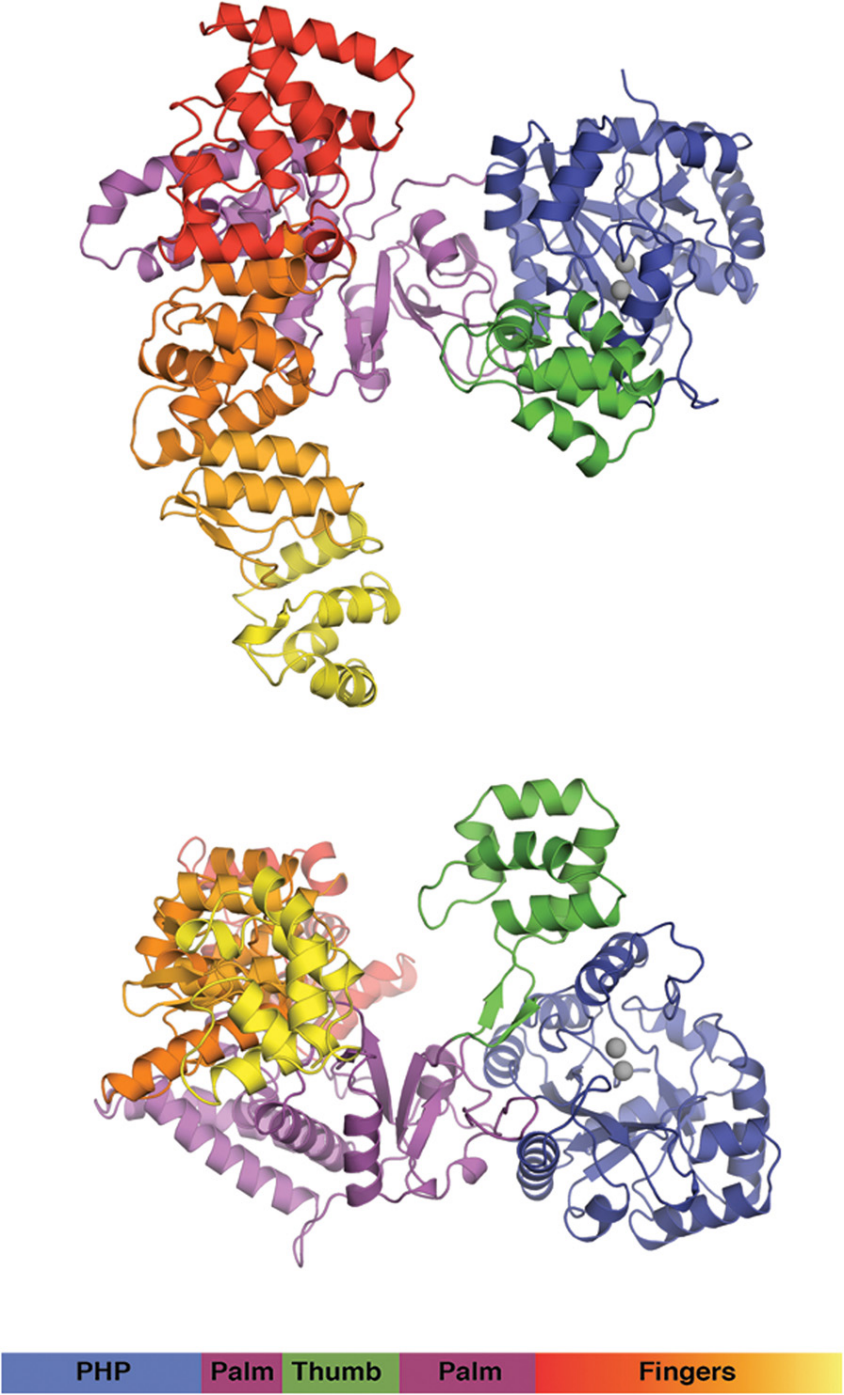
**Structure of 3mPHP.** The figure shows two orthogonal views of the 3mPHP structure determined at 2.9 Å resolution. The Pol III domains are coloured individually and the bound Zn^2+^ ions are shown as grey spheres.

**Figure 4:**
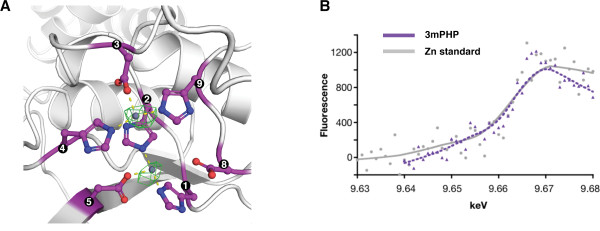
**Metal binding by the 3mPHP mutant.** (**A**) Detail of the 3mPHP active site showing two peaks on the anomalous difference map contoured at 3.5 sigma shown in green. The two modelled Zn^2+^ ions are shown as spheres. Yellow dashed lines represent the distance (2.0 to 2.1 Å) between the side chains of the metal-binding residues and the centre of the two peaks. The (**B**) X-ray fluorescence scan of a zinc standard solution (grey) and of a 3mPHP protein sample (purple).

We crystallized 3mPHP under similar conditions to the wild-type protein, and the structure was determined by molecular replacement and refined to 2.9 Å resolution (Table 2; Figure 3). The structure presents the typical cupped right hand conformation of a DNA polymerase (Figure 3), with well-defined Fingers and Palm and a Thumb that sits on top of the PHP domain. The 3mPHP structure is virtually identical to that of the wild-type protein [5], with a root square mean deviation of 0.92 Å over 756 aligned Cα atoms. However, 3mPHP revealed additional features in the electron density map at the centre of PHP active site (Figure 4A). Two strong peaks (5.0 σ and 6.1 σ) that stand out in the anomalous difference map calculated using X-rays of 1.000 Å wavelength, indicate the presence of two metals bound to the active site of the PHP domain, even though no metals were added to the crystallisation conditions. The positions of the two metal ions in the 3mPHP structure are essentially identical to the metal positions in other PHP domain structures, including *T. aquaticus* Pol III [6]. A third metal ion often found in canonical PHP domains is not observed. This third metal ion is not seen in the original Taq structure either, and its absence in the 3mPHP structure may be due to the lack of the cysteine at position 7 and the glutamate at position 5 in 3mPHP. Attempts to crystallise the 4mPHP and 5mPHP variants were unsuccessful.

**Table 2:** X-ray diffraction data processing and model refinement statistics

	***E. coli*3mPHP**
***Data collection***	
**Beamline**	ALS 8.2.2
**Wavelength (Å)**	1.000
**Space group**	P2_1_2_1_2_1_
**Unit cell** (a,b, c in Å)	82.43, 98.94, 139.88
**Resolution (Å)**	46.94 - 2.90
**CC1/2**	99.4 (56.5)
**Rsym**	0.132 (1.030)
**Mean (I)/(sigI)**	14.1 (2.1)
**Completeness**	100.0 (100.0)
**Multiplicity**	7.3 (7.4)
***Refinement***	
**Rwork / Rfree**	19.4 / 24.5
**No. atoms**	7190
protein	7147
waters	20
ligands	23
**RMS(bonds) / RMS (angles)**	0.003 / 0.733
**Ramachandran favored (%)**	95.0
**Ramachandran outliers (%)**	0.4
**Molprobity Clashscore**	18.02

To identify the metals observed in the 3mPHP crystal structure, we analysed a protein sample treated in an identical manner to the solution used for crystallisation using X-ray fluorescence. We found that the X-ray fluorescence spectrum of our sample very closely resembles that of a zinc standard (Figure 4B). The two metal ions in the 3mPHP structure were, therefore, modelled as zinc.

### Re-introduction of the catalytic residues in the PHP domain does not result in exonuclease activity

**Figure 5:**
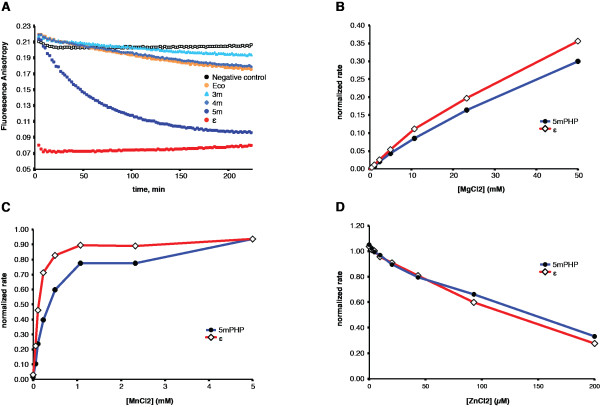
**Restoration of metal-binding in *E. coli *Pol III does not induce exonuclease activity.** (**A**) *E. coli* Pol III wild-type, 3mPHP and 4mPHP mutants show virtually no exonuclease activity in our measurements, as opposed to *E. coli* Pol III **ϵ** subunit that shows very robust activity under the same experimental conditions. The 5mPHP mutant shows some exonuclease activity, that is ~30-fold lower than that of the *E. coli* Pol III **ϵ** subunit. However, the metal-dependence of this activity is identical to that of the **ϵ** subunit. For both protein preparations, the exonuclease activity is stimulated by Mg^2+^ (**B**) and Mn^2+^, but is inhibited by Zn^2+^ (**C**; 0.3 mM MnCl_2_ background), suggesting that the observed activity for the 5mPHP preparation is due to contamination by **ϵ** subunit.

As the crystal structure of the 3mPHP mutant shows that it has acquired metal binding in its PHP domain, we wondered if our mutants had also acquired exonuclease activity. To measure exonuclease activity, we developed an assay based on fluorescently labelled DNA. By measuring the decrease in fluorescence anisotropy, we could readily follow the degradation of DNA molecules in real-time (see Experimental Procedures). As shown in Figure 5, ssDNA is efficiently degraded by the exonuclease ϵ, which is the *bona fide* proofreading subunit of the DNA polymerase III holoenzyme. In contrast, wild-type *E. coli* Pol III shows only a very low level of exonuclease activity. Likewise, the mutants 3mPHP and 4mPHP do not show increased 3′-5′ exonuclease activity relatively to the wild-type protein in the presence of any of the metals tested (Zn^2+^, Ni^2+^, Mn^2+^, Co^2+^, Cu^2+^; Figure 5 and data not shown), which may be explained by the fact that these mutants do not have all 5 missing residues restored. The quintuple mutant 5mPHP does have all residues for metal binding and did show increased exonuclease activity, that is ~30 fold lower than that for the exonuclease ϵ alone. The 5mPHP mutant however shows some impurities present in the purified protein, raising the possibility that the activity could be caused by a contaminating exonuclease, most likely ϵ subunit which is known to bind Pol III with nanomolar affinity. To verify this hypothesis we further investigated the metal-dependence of the observed exonuclease activity.

*In vivo,* the exonuclease activity of the ϵ subunit is dependent on the binding of Mg^2+^, while *in vitro* this activity is enhanced by replacing the Mg^2+^ with Mn^2+^[26]. As shown in Figure 5B and 5C, we observed that the exonuclease activity present in the 5mPHP sample is stimulated by both metals and that the activity with Mn^2+^ is higher than with Mg^2+^, as with the ϵ subunit. On the other hand, we found that Zn^2+^ has an inhibitory effect (Figure 5D). This is in contrast to the *T. thermophilus* Pol IIII exonuclease activity which was found to be Zn^2+^ dependent [12]. It is therefore likely that the exonuclease activity measured in *E. coli* Pol III preparations, and especially in 5mPHP preparations, is caused by an impurity in the protein sample, most likely endogenous *E. coli* ϵ subunit.

### The PHP domain provides stability to the polymerase

**Figure 6:**
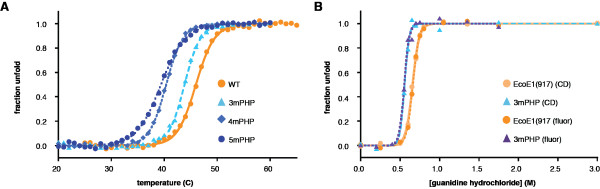
**Mutations at the PHP domain decrease the overall stability of *E. coli *Pol III.** The thermal and chemical stability of Pol III decreases gradually with the number of mutations introduced at the PHP domain, as measured by (**A**) temperature melt followed by circular dichroism or (**B**) through chemical denaturation using guanidine-hydrochloride titrations followed by circular dichroism and tryptophan fluorescence. Pol III shows apparent two-state unfolding.

While the PHP domains of the Pol III protein from the α-, β-, and γ-proteobacteria do not have a complete set of metal-coordinating residues, the PHP domain itself is always present and shows clear conservation within this clade of bacteria. We therefore wondered if the PHP domain in this group of Pol III proteins might fulfil another function. To further investigate this, we performed a series of unfolding experiments using circular dichroism and tryptophan fluorescence (Figure 6A). We find that the melting temperature (T_m_) of the protein decreases with the number of mutations introduced, from 46.0 to 39.4°C, indicating that the mutations in the PHP domain affect the overall stability of the protein.

Interestingly, the unfolding of the protein appears to be a cooperative event as indicated by the single sigmoidal curve obtained by both melting temperature experiments monitored by circular dichroism and tryptophan fluorescence. Taken together, the two curves reveal that the secondary structure (as reported by circular dichroism) and tertiary structure (as reported by tryptophan fluorescence) of Pol III break down simultaneously in a two-state transition. The same single step unfolding was observed when the unfolding of the protein with the chemical denaturant guanidinium chloride as measured by circular dichroism (Figure 6B). Here too, we find that the protein apparently unfolds in a single step, with the 3mPHP mutant being less stable than the wild-type protein.

It is rather surprising that a protein as large as this Pol III catalytic construct (100 kDa) apparently unfolds in single step. Large multi-domain proteins often exhibit intermediate states of unfolding due to the sequential unfolding of the individual domains [27,28]. The single step unfolding of *E. coli* Pol III indicates that the different domains unfold cooperatively. The correlation between the number of experimental mutations at the PHP domain and decrease in the T_m_ values of Pol III highlights how the PHP domain is structurally integrated with the rest of the polymerase. Our data therefore suggest that the PHP domain plays a crucial non-enzymatic role in stabilising the entire structure of Pol III.

### Mutations at the PHP domain modulate polymerase activity

**Figure 7:**
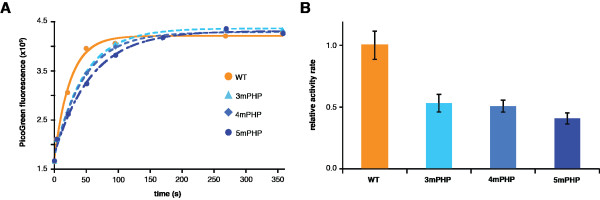
**Mutations at the PHP domain decrease Pol III polymerization activity.** (**A**) Production of dsDNA was monitored by the intercalating dye PicoGreen. *E. coli* PHP mutants show substantially reduced polymerization activity. The decrease in activity correlates with the number of mutations introduced in the PHP domain. The relative polymerization rates of WT *E. coli* Pol III and the PHP mutants are shown in (**B**).

Having established a role for the PHP domain in the structural integrity of C-family polymerases, we set about probing the influence of this domain on enzymatic function. We measured the polymerase activity of the wild-type *E. coli* Pol III crystallization construct and also the three PHP mutants. Analogous to the earlier stability trend, the activity of the polymerases decreased as the number of mutations in the PHP domain was increased (Figure 7). For 3mPHP and 4mPHP, the polymerization rate decreased to roughly half that of the WT protein, and an additional decrease was observed for 5mPHP. These decreases cannot be simply attributed to the lower stabilities of the mutant proteins, as the polymerization assays were performed at 20°C, well below the denaturation onset temperature (30°C) for even the least stable of the mutants (Figure 6A). The observed decreases in polymerase activity for the mutants show that the PHP domain helps tune the dynamics of Pol III for catalysis. Given the stabilizing role of the PHP domain, its contribution to function is perhaps expected, but the distance — more than 30 Å — between the polymerase active site in the Palm domain and the mutated residues in the PHP domain is evidence of an intimate conformational coupling between these domains and underscores the critical importance of the PHP domain as a structural scaffold within Pol III.

## Conclusions

Our results emphasize the fact that bacterial replicative polymerases have maintained the structure of PHP domains that have lost metal-binding residues. The extent of this conservation is remarkable, as it has survived the billions of years of evolution subsequent to the split between those bacterial species that retained metal-binding residues (with presumed retention of the enzymatic activity) and those that have lost it, of which *E. coli* is the primary example. Biochemical studies have indicated that the association between the ϵ and α subunits involves the polymerase PHP domain and the C-terminal tail of ϵ [29,30]. The correlation between loss of PHP domain activity and the presence of an ϵ homologue in the corresponding genome suggests that the strict structural conservation of the PHP domain might arise from the necessity to precisely position the active site of the *trans* exonuclease near the PHP cleft. Furthermore, the substantial decrease in polymerization activity and global stability of our PHP mutants clearly indicates that the scaffolding role of the PHP domain goes beyond the positioning of *trans* exonucleases. It suggests that the PHP domain is a major structural element for the stabilization of Pol III and plays a key role in the provision of optimal Pol III activity.

## Methods

### Protein sequence analysis

Replicative C-family polymerase sequences were retrieved using Protein BLAST at the Bioinformatics Toolkit hosted by the Max-Planck Institute for Developmental Biology (http://toolkit.tuebingen.mpg.de/prot_blast). The input sequence was *E. coli* Pol III, the database was nr_bac70 (the bacterial sequences in NCBI protein database filtered to a maximum of 70% identity), and all other parameters were defaults. The resulting alignment was manually edited to include only sequences identified as Pol III with high confidence. For easier visualization, this alignment was reduced to its 47 most diverse sequences using AlignmentViewer (http://toolkit.tuebingen.mpg.de/alnviz). To broaden the scientific interest of the polymerase sample set, some sequences in the reduced alignment were replaced with homologs from organisms of medical, industrial and/or historical interest. Because the alignment from Protein BLAST included only those sequence regions scored as homologous to *E. coli* Pol III, the complete sequence for each polymerase chosen for the sample set was retrieved using its GID number and RetrieveSeq (http://toolkit.tuebingen.mpg.de/gi2seq). These sequences were aligned using MAFFT L-INS-i (http://mafft.cbrc.jp/alignment/server/index.html), and a neighbor-joining phylogenetic tree was generated using the MAFFT server (settings: all gap-free sites, WAG substitution model, estimate heterogeneity among sites, bootstrap resampling = 100).

Proteins homologous to the DNA polymerase ϵ exonuclease subunit were gathered by (1) retrieving from the Conserved Domains Database [31] the sequence alignment of all 232 proteins used to define the conserved domain PRK05711 (DNA polymerase III subunit epsilon; Provisional) and (2) performing a blastp search (http://blast.ncbi.nlm.nih.gov/Blast.cgi?PROGRAM=blastp&BLAST_PROGRAMS=blastp&PAGE_TYPE=BlastSearch) limited to the organisms included in the Pol III sequence analysis. All sequences with expect scores equal to or better than that of the worst scoring sequence annotated as an ϵ exonuclease were kept and aligned using MAFFT L-INS-i, and a phylogenetic tree was generated using FastTree 2.1.7 [32]. All sequence alignments were visualized using Jalview 2.8 [33] and all trees with FigTree 1.3.1 and 1.4 (http://tree.bio.ed.ac.uk/software/figtree/).

### Mutagenesis and protein purification

Mutations were introduced in a truncated version of *E. coli* DNA polymerase III α subunit (residues 1-917) [5] according to Table 1 using the Quikchange kit from Stratagene. All *E. coli* Pol III constructs included a N-terminal His6 tag, followed by a Prescission protease cleavage site and were expressed and purified using a protocol based on the method described in [5] , with the addition of a Ni-resin chromatography purification step after cell lysis.

### Crystallisation and structure determination

Crystals of *E. coli* 3mPHP were grown using the hanging drop vapour diffusion method under conditions similar to the WT version [5] albeit with slightly lower precipitant concentration. Concentrated protein at 10-15 mg/ml was mixed with 15%-20% PEG3350, 0.2-0.4 M NaH_2_PO_4_, 100 mM HEPES pH 7.5. Crystals were frozen in mother liquor including 20% glycerol. The structure was solved by molecular replacement using the WT structure [5] as search model in PHASER [34]. The model was further improved by multiple rounds of manual rebuilding in COOT [35] and refinement in REFMAC [36] and phenix.refine [37]. Coordinates and structure factors for 3mPHP were deposited in the Protein Data Bank data with the accession code 4JOM.

### Polymerase preparation for biochemical assays

Polymerase stock solutions were thawed from storage at -80°C, diluted with a concentrated monomerization buffer (supplemental concentrations after addition: 15% glycerol, 20 mM HEPES pH 7.4, 100 mM NaCl, 0.1 mM TCEP), and monomerized by incubation overnight at 15°C. Polymerases were confirmed to behave as monomers by size-exclusion chromatography on an S200 SMART column. For storage over several days at -20°C, a high-glycerol buffer was added as a cryoprotectant (supplemental concentrations after addition: 50% glycerol, 20 mM TAPS pH 8.5, 100 mM NaCl, 2.25 mM TCEP).

### Exonuclease activity assays

Activity was detected using a novel, real-time fluorescence anisotropy assay. Single-stranded DNA labeled on its 3′-end was purchased from IDT (TAGGACAGTTCACGCTTCTTGG-TAMRA). Exonuclease activity at the 3′-end of the DNA first cleaves the TAMRA label from the DNA in a reaction with apparent first-order kinetics. Reactions (150 μL) were initiated by adding 2.8 μM protein to aliquots of 50 nM labeled DNA (buffer: 15% glycerol, 0.2 mg/mL BSA, 20 mM TAPS pH 8.5, 100 mM K glutamate, 10 mM MgCl_2_, 10 mM βME) in opaque black 96-well plates and monitored using a Perkin-Elmer Victor3 fluorescence plate reader with a 535/30 nm excitation filter, 595/60 nm emission filter, and an averaging time of 1 sec. The metal-dependence of the exonuclease activity was tested using an assay based on [26]. The activity was tested in a buffer containing 40% glycerol, 1 mg/ml lysozyme, 20 mM HEPES at pH 7.5, 100 mM K glutamate and 0.5 mM TCEP. The Zn^2+^ titration was performed in the presence of 0.3 mM MnCl_2_.

### Determination of melting temperature

Samples (1.5 mL) were individually subjected to temperature titrations with 1°C intervals separated by 1 minute equilibration periods. Data collection occurred in a sealed 4-mL Hellma quartz cuvette using a FluoroMax-3 (Jorbin Yvon Horiba) fluorometer with a Wavelength Electronics Model LFI-3751 temperature controller. Excitation occurred at 280 nm (slit width 3.5 nm), and emission scans were collected from 295 to 397 nm (slit width 7 nm) in 2 nm increments with 0.5 s of integration time and then converted into scan centres of mass. KaleidaGraph (Synergy Software) was used for curve fitting to a standard temperature melt equation. Details on data reduction and curve fitting are provided in Additional file 3.

### Chemical denaturing

An individual sample (1 mL) was prepared for each data point, and all samples were allowed to equilibrate at 25°C overnight (roughly 18 h). Samples were held in a 4-mL Hellma quartz cuvette during analysis. Circular dichroism was measured in kinetic mode on a Circular Dichroism Spec 410 (AVIV Biomedical) at 226 nm using 60 separate 1-sec reads. The reads for each sample were averaged and normalized by conversion into units of mean residue ellipticity. After circular dichroism analysis, a FluoroMax-3 (Jorbin Yvon Horiba) was used to assay tryptophan fluorescence. Excitation occurred at 280 nm (slit width 2 nm), and emission scans were collected from 295 to 397 nm (slit width 4 nm) in 2 nm increments with 0.5 s of integration time. Each scan was reduced to its centre of mass. Increasing Gdn•HCl caused a non-linear shift of the scan centres of mass toward longer wavelengths. To simplify curve fitting, the points corresponding to the highest Gdn•HCl concentration (3 M) samples were omitted during data analysis. KaleidaGraph (Synergy Software) was used for curve fitting to a standard denaturant melt equation. Details on data reduction and curve fitting are provided in Additional file 3.

### DNA polymerization assay

DNA polymerization was monitored by fluorescence intensity using a slightly modified version of the standard PicoGreen-based quench assay [38-40] in 96-well format. The substrate was a DNA primer-template complex generated by annealing two oligomers: Template 5′-TTGTGGGTAGATAAATACAGACCTAAGTCCTTGAATGCCGCGTGCGTCCC and Primer 5′-GGGACGCACGCGGCATTCAAGGA. The assays were performed at 250 nM polymerase, 7.5 nM DNA and 50 μM of each dNTP. The reaction buffer included 20 mM HEPES at pH 7.5, 100 mM NaCl, 0.2 mg/ml BSA, 3 mM MgCl_2_ and 0.5 mM TCEP. Time point samples were taken until the reactions reached completion. After quenching the last sample, 90 minutes were allowed for fluorescence development before data acquisition on a Perkin-Elmer Victor3 fluorescence plate reader using a 535/30 nm excitation filter, 595/60 nm emission filter, and an averaging time of 1 sec. To achieve stable readings, samples were scanned 9 times, and the last 5 reads were used for curve fitting. Rate constants were fit in KaleidaGraph using a standard exponential function.

## Competing interests

The authors declare that they have no competing interests.

## Authors’ contributions

TB and JG designed and performed research, analyzed data, and drafted the manuscript. BK designed and performed research and analyzed data. JA and AP performed research. MO’D designed research and analyzed data. JK and MHL designed research, analyzed data, and drafted the manuscript. All authors read and approved the final manuscript.

## Supplementary Material

Additional file 1 **Sequence alignment of replicative C-family DNA polymerases.** The full alignment of replicative C-family DNA polymerase sequences. Trees in Figure 2 were generated based on this alignment. Click here for file

Additional file 2 **Sequence alignment of DEDD exonucleases.** The full alignment of DEDD exonuclease sequences obtained from all species represented in the replicative C-family DNA polymerase sequence alignment. Click here for file

Additional file 3 **Supplementary methods.** The data reduction and curve fitting procedures. Click here for file
