# Supplementary material for: A structural role for the PHP domain in E. coli DNA polymerase III
Source: BMC Struct Biol. 2013 May 14;13:8. doi: 10.1186/1472-6807-13-8 (PMC3666897; doi:10.1186/1472-6807-13-8)
Supplement: Additional file 3 — Supplementary methods. The data reduction and curve fitting procedures. [file 1472-6807-13-8-S3.pdf]

Supplementary methods

DNA polymerization assay

DNA polymerization rate constants were fit in KaleidaGraph using a standard exponential function:

$$F = F_\infty - Ae^{-kt}$$

Equation 1. Exponential fit of DNA polymerization data.

Fluorescence, F, was monitored as a function of time, t. F_∞ is the fluorescence at time infinity (i.e. the fluorescence were the reaction to reach completion). A is the fluorescence amplitude increase from reaction initiation to completion. The parameter of interest, k, is the reaction constant, which is proportional to the rate of DNA polymerization.

Denaturant and temperature melts

Circular dichroism reads collected every 60 seconds overnight for each sample were averaged and normalized by conversion into units of mean residue ellipticity Equation 2:

$$[\Theta] = \frac{\Psi}{10\ell cN}$$

Equation 2. Conversion of circular dichroism signal to mean residue ellipticity.

$[\Theta]$ is the mean residue ellipticity, which is a function of circular dichroism signal, Ψ, divided by path length in cm, ℓ, molar protein concentration, c, and the number of amino acid residues in the protein, N.

Tryptophan fluorescence emission scans were reduced to their centres of mass using Equation 3:

$$\langle\lambda\rangle = \frac{\sum_{i=1}^{n} F_i \lambda_i}{\sum_{i=1}^{n} F_i}$$

Equation 3. Tryptophan fluorescence scan centre of mass determination.

$\langle\lambda\rangle$ is the scan center of mass (i.e. the fluorescence emission intensity-weighted average scan wavelength). F_i is the fluorescence intensity corresponding to wavelength λ_i.

Denaturant melt data from circular dichroism and tryptophan fluorescence experiments were fit to a standard denaturant melt equation with variables for the native state baseline, unfolding transition and denatured state baseline.

$$S = \frac{\alpha_N + \beta_N[D] + (\alpha_D + \beta_D[D])e^{\frac{m([D]-c_m)}{RT}}}{1 + e^{\frac{m([D]-c_m)}{RT}}}$$

Equation 4. Raw denaturant melt curve fitting.

S is the protein signal, from either circular dichroism or tryptophan fluorescence analysis, as a function of $[D]$, denaturant concentration. α_N and α_D are the signals of the native and denatured states, respectively, of the protein in the absence of denaturant. $\beta_N$and β_D are the slopes of the baselines for the native and denatured states, respectively. m is the slope of the unfolding transition. R and T are the ideal gas constant and temperature, respectively. The parameter of interest, c_m, is the denaturant concentration at which the folded and unfolded states of a protein are equally populated at equilibrium.

To overlay the circular dichroism and fluorescence data, the output from fitting the raw denaturant melt data was normalized using Equation 5, which was generated by inserting the native and denatured baseline equations into the definition of the fraction denatured.

$$f_D = \frac{\alpha_N + \beta_N[D] - S}{\alpha_N + \beta_N[D] - (\alpha_D + \beta_D[D])}$$

Equation 5. Normalization of denaturant melt data.

The fraction of denatured protein, f_D, in terms of the observed signal, S, and the signal baselines for the native, S_N, and denatured, S_D, states of the protein. All symbols not defined here are the same as in Equation 4.

The normalized denaturant melt data was fit in KaleidaGraph using the simplified Equation 6:

$$S = \frac{1}{1 + e^{\frac{-m([D] - c_m)}{RT}}}$$

Equation 6. Normalized denaturant melt curve fitting.

For normalized denaturant melt data, the fitting equation is simpler because the lower (native) and upper (denatured) baselines are 0 and 1, respectively. The symbols used are the same as in Equation 4.

The same equations were used to fit the temperature melt data, substituting in Equations 4, 5 and 6 the denaturant concentration [*D*] for the temperature *T* and c_m for the melting temperature T_m.
